# Potential Unlocking of Biological Activity of Caffeic Acid by Incorporation into Hydrophilic Gels

**DOI:** 10.3390/gels10120794

**Published:** 2024-12-04

**Authors:** Monika Jokubaite, Kristina Ramanauskiene

**Affiliations:** 1 Department of Drug Chemistry, Faculty of Pharmacy, Lithuanian University of Health Sciences, Sukileliai Avenue 13, LT-50162 Kaunas, Lithuania; 2 Department of Clinical Pharmacy, Faculty of Pharmacy, Lithuanian University of Health Sciences, Sukileliai Avenue 13, LT-50162 Kaunas, Lithuania; kristina.ramanauskiene@lsmu.lt

**Keywords:** caffeic acid, poloxamer, carbomer, hydrogel, antimicrobial

## Abstract

Caffeic acid, a phenolic compound with antioxidant and antimicrobial properties, shows promise in the dermatological field. The research aimed to incorporate caffeic acid into hydrophilic gels based on poloxamer 407, carbomer 980, and their mixture in order to enhance its biological activity. Different gel formulations were prepared using different concentrations of these polymers to optimize caffeic acid release characteristics. The results showed that increasing the concentration of polymeric materials increased the viscosity and slowed down the release of caffeic acid. The antioxidant and antimicrobial activities of the gels were assessed. The results confirmed the potential of hydrophilic gels as delivery systems for caffeic acid, with formulations showing antimicrobial activity against Gram-positive *Staphylococcus aureus* bacteria and antifungal activity against *Candida albicans* fungus. This study provides a perception of the development of new semi-solid caffeic acid-based formulations for therapeutic and cosmetic applications.

## 1. Introduction

The skin is the largest protective organ in the human body, and its pathologies are caused by various disorders such as inflammatory diseases, dehydration, and even skin cancer. For effective treatment, the search for natural active components is increasingly prevalent in scientific research strategies [1]. One of the most commonly used antioxidants for the skin is ascorbic acid. However, it should be noted that this acid is highly unstable, which drives the search for new components with potential antioxidant properties [2]. Many phenolic acids have therapeutic effects—one of them is caffeic acid (3,4-dihydroxycinnamic acid), which is synthesized in many medicinal plant species, plant-based food sources, and bee products like propolis [3,4,5]. The most prevalent hydroxycinnamic acids found in raw plant materials are caffeic acid, ferulic acid, and *p*-coumaric acid. The prevalence and biological properties of caffeic acid have prompted researchers to investigate its biological effects and potential applications in the pharmaceutical, cosmetics, and food industries [6]. Caffeic acid (CA) exhibits antimicrobial effects (against *E. coli*, *P. aeruginosa*, *B. cereus*, *M. luteus*, *S. aureus*, *L. monocytogenes*, *Candida albicans*) [7,8], antioxidant properties [9], immunomodulatory [10,11], antidiabetic [12,13], antiviral [14,15], anticancer, and anti-inflammatory activities [16,17]. This phenolic acid provides protection against ROS-induced damage to DNA, lipids, proteins, and other molecules [18]. The antioxidant potential of caffeic acid has been described in emulsions composed of 30% sunflower oil in water (O/W) and 20% water in sunflower oil (W/O) at pH 5.4 [19,20]. In vitro studies have shown that caffeic acid and its derivatives can protect skin fibroblasts from UVA-induced oxidative stress by activating the intracellular antioxidant system [9]. Caffeic acid derivatives protect the skin from UV-induced oxidative damage and help prevent UV-induced photoaging [21]. Topically applied caffeic acid (30 mg kg^−1^) prevented the decrease in moisture and hydroxyproline levels, as well as the thickening of the epidermis in test mice [22]. In vitro studies have demonstrated the antiviral activity of caffeic acid against Herpes simplex virus types 1 and 2, as well as influenza A virus [23,24]. In vivo studies have shown that film dressings containing caffeic acid, which possess controlled-release properties, can be excellent candidates for skin wound healing—the growth of new epidermis towards the center of the wound was observed, resulting in a reduction in wound area [25]. It can be stated that due to its multiple activities, caffeic acid is a promising active ingredient for protective effects in cosmetic and therapeutic dermatological formulations [25,26]. The antimicrobial, antioxidant, and anti-inflammatory properties of caffeic acid can contribute to mitigating photoaging and protecting the skin against UV-induced damage.

When designing semi-solid preparations with caffeic acid (CA), it is important to select an appropriate carrier for the active substance. The chosen base should ensure the stability of the active substance, its release, distribution on the skin, and its transport into skin layers. It should be noted that the incorporation of caffeic acid into nanoparticles within a gel ensured prolonged CA antioxidant activity in the upper epidermis [27]. It is known that stratum corneum hydration is one method to improve the penetration of medicinal substances through the lipid barrier [28]. One way to moisturize the skin is to use gelled semi-solid hydrophilic systems. It is relevant to incorporate caffeic acid into semi-solid hydrophilic systems, such as gels, which have a non-greasy texture, release the active substance, adhere well to the skin or mucosal surface, and are easy to apply and wash off. Gels can be produced with both polar and non-polar active substances. They can be used as systems for controlling the release of the active substance. Due to the possibility of incorporating various active substances, their favorable sensory properties, and other advantages, gels are a suitable semi-solid pharmaceutical form for incorporating caffeic acid. Considering the limited solubility of caffeic acid, poloxamer 407 was chosen as one of the gelling agents, as it is an effective carrier that enhances the solubility of hydrophobic substances. Poloxamers swell and gel in water, thus prolonging the release of the active ingredient from the pharmaceutical form. The pH of poloxamers in aqueous solution ranges from 5.0 to 7.5 [29]. Maddalena Sguizzato et al. applied P407 SNP for incorporating CA into gels; this gelling agent forms aggregates in water, creating micelles, thereby increasing the solubility of CA [27].

Carbomers are known as environmentally friendly polymers with bioadhesive properties and excellent organoleptic properties. They are used in the production of skin, oral mucosal, eye, and vaginal gels [30], making them suitable candidates for modeling controlled drug delivery systems. Carbomer 980, a bioadhesive polymer, enhances the gel strength of temperature-sensitive hydrogels. Neutralization with sodium hydroxide or other bases ionizes carboxyl groups, leading to partial polymer chain separation and irreversible agglomerate formation [31]. Typically used at concentrations of 0.5–3%, Carbomer 980 can also be combined with P407 gels to form composite hydrogels, whose properties depend on the physicochemical interactions of the components [32]. The aim of the study is to evaluate the biological activity of caffeic acid incorporated into gelified formulations and to perform an in vitro biopharmaceutical assessment. By incorporating caffeic acid into a hydrogel system with controlled release characteristics, we seek to explore its applicability for topical pharmaceutical formulations. This research may contribute to the development of cosmetic and pharmaceutical products, providing new insights into the application of natural components in the creation of semi-solid formulations.

## 2. Results

### 2.1. Modeling of Experimental Gels with Caffeic Acid

Different compositions of experimental gels were prepared using Poloxamer 407 (12–18%) and Carbomer 980 (0.5–1%) as gelling agents, along with 2% caffeic acid. The compositions of the gels with caffeic acid are presented in Table 1. Homogeneous gels were produced, exhibiting a characteristic light yellowish color typical of gels.

### 2.2. Viscosity Dependencies of the Polymer Bases

**Figure 1:**
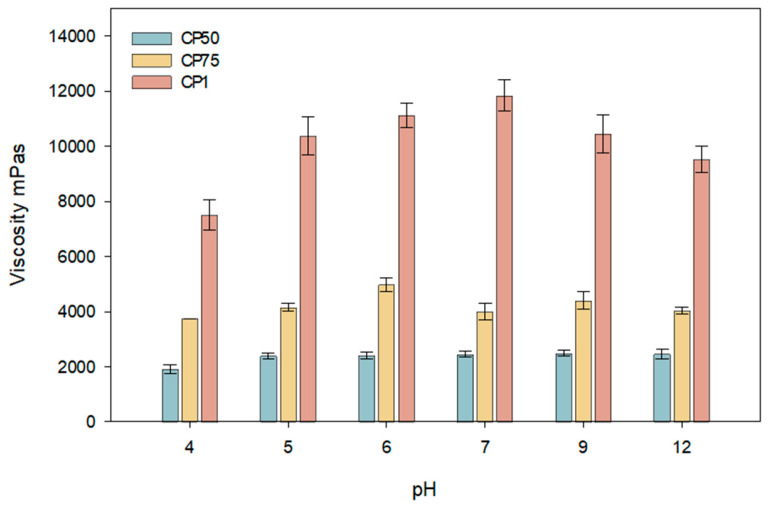
Viscosity dependence of carbomer gels with caffeic acid on different pH ranges (CP50, CP75, CP1; *n* = 3, mean ± SD).

During the modeling of carbomer gels with varying concentrations, the changes in dynamic viscosity at different pH values were evaluated. The results of these studies are presented in Figure 1. Formulations with Carbomer 980 pH before neutralizing with NaOH reached 3.48, 3.3, and 3.1 (when Carbomer 980 is 0.5, 0.75, and 1%, respectively).

This study determined that the viscosity of carbomer gels depends on the formulation’s pH and polymer concentration (Figure 1). The results of the study indicate that the viscosity of carbomer gels containing caffeic acid is influenced by both the pH of the formulation and the polymer concentration. Higher viscosity was observed at pH 7 when the polymer concentration was 1%. The highest viscosity was achieved at pH 7 with a polymer concentration of 1%. Formulations with concentrations between 0.5% and 0.75% exhibited similar viscosity values in the pH range of 5 to 6. A decrease in viscosity was observed for all formulations at a pH value of 12. This reduction may be associated with diminished intermolecular interactions and structural stability. The results suggest that optimizing the viscosity and stability of carbomer gels is most effective in slightly acidic or neutral conditions (pH 5–7), which is beneficial for applications in the pharmaceutical and cosmetic industries. For further research, a skin-neutral pH was chosen when the pH was 5.5.

**Figure 2:**
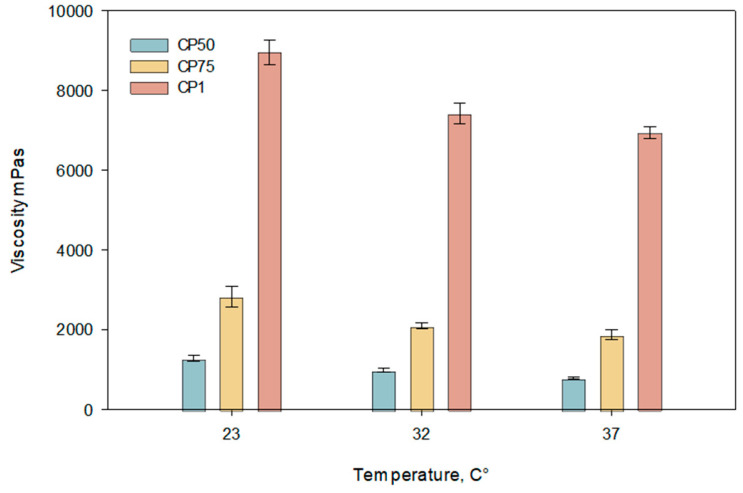
Temperature dependence of viscosity of carbomer gels containing caffeic acid at pH 5.5 ± 0.3 (CP50, CP75, CP1). Measurements were performed at 23 °C, 32 °C, and 37 °C (*n* = 3, mean ± SD).

The results of the study indicate a decreasing trend in viscosity with increasing temperature (Figure 2). Viscosity values significantly decreased as the temperature rose from 23 °C to 32 °C. At 37 °C, a statistically significant reduction in viscosity was observed compared to that at 23 °C (*p* < 0.05). The graph shows that formulations with a carbomer concentration of 1% exhibited the highest viscosity, while lower concentrations (0.75% and 0.5%) displayed proportionally lower viscosity values across all tested temperatures.

**Figure 3:**
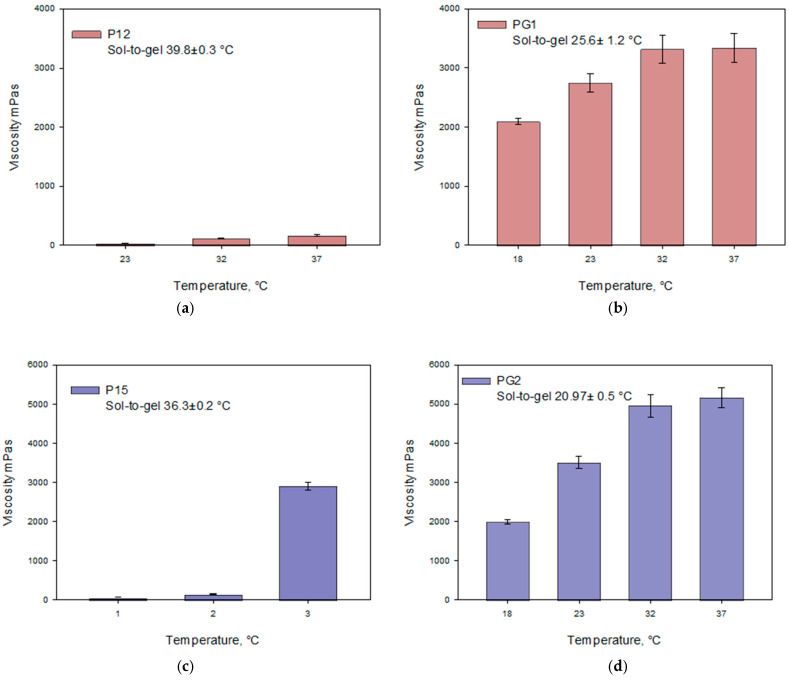

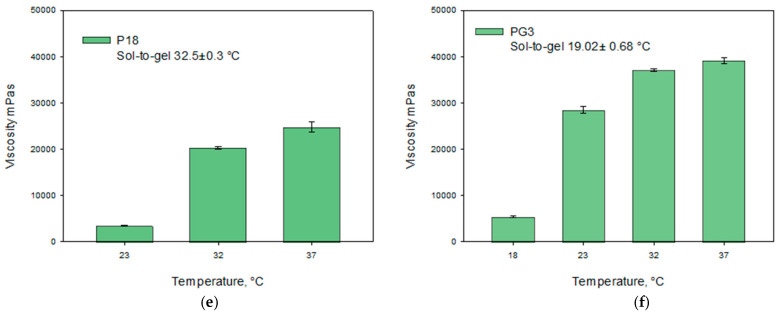
Viscosity dependence on temperature of Poloxamer 407 and Poloxamer 407 + Carbomer 980 gels with caffeic acid (pH 5.5 ± 0.3) at different polymer concentrations: (**a**) P12, (**b**) PG1, (**c**) P15, (**d**) PG2, (**e**) P18, (**f**) PG3 (*n* = 3, mean ± SD).

For temperature-sensitive gels made with a poloxamer base, an increase in temperature directly influenced an increase in viscosity values. A sharp increase in viscosity was observed when the gel temperature rose from 23 °C to 32 °C, forming semi-solid gelled systems. As the temperature continued to increase, the viscosity values of the gels continued to rise, although to a lesser extent, which may be related to the gelation temperature of the gels. The data presented in Figure 3 indicate that as the concentration of poloxamer in the gels increases, the gelation temperature decreases. The study results demonstrate that the viscosity values of the gels increased with the rising concentration of poloxamer.

The selected combination of polymers enhanced the gelation properties of the modeled systems. The formulated gels are thermosensitive, with their viscosity increasing as the temperature rises. When the gels contain poloxamer, their viscosity significantly depends on the amount of carbomer incorporated. Statistically significant lower viscosity was observed in gels P12–P18 compared to gels PG1–PG3. The viscosity values of gels PG1–PG3 differed significantly at temperatures of 18 °C and 23 °C (*p* < 0.05).

### 2.3. Evaluation of the Solubility of Caffeic Acid and In Vitro Release Study of Caffeic Acid from Experimental Gels

**Figure 4:**
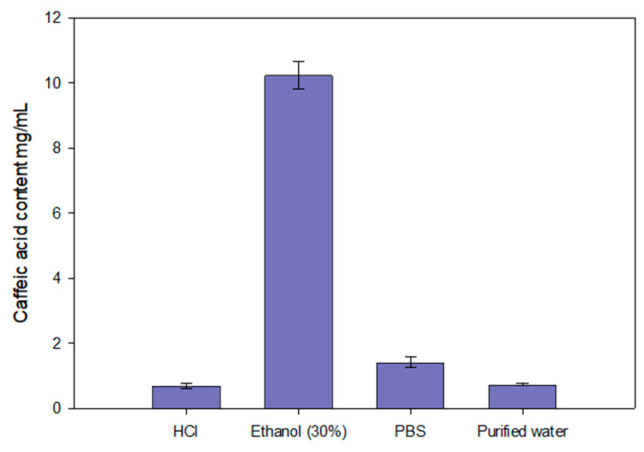
Solubility of caffeic acid in purified water, HCl (pH = 1.2), PBS (pH = 6.8), and 30% (*v/v*) ethanol (*n* = 3, mean ± SD).

The data presented in Figure 4 show that the highest amount of caffeic acid dissolved in a 30% (*v/v*) ethanol solution. The addition of ethanol significantly improved the solubility of caffeic acid compared to other tested solvents (*p* < 0.05). Consequently, 30% (*v/v*) ethanol was selected as the acceptor medium for the in vitro release study of caffeic acid from the modeled gels.

**Figure 5:**
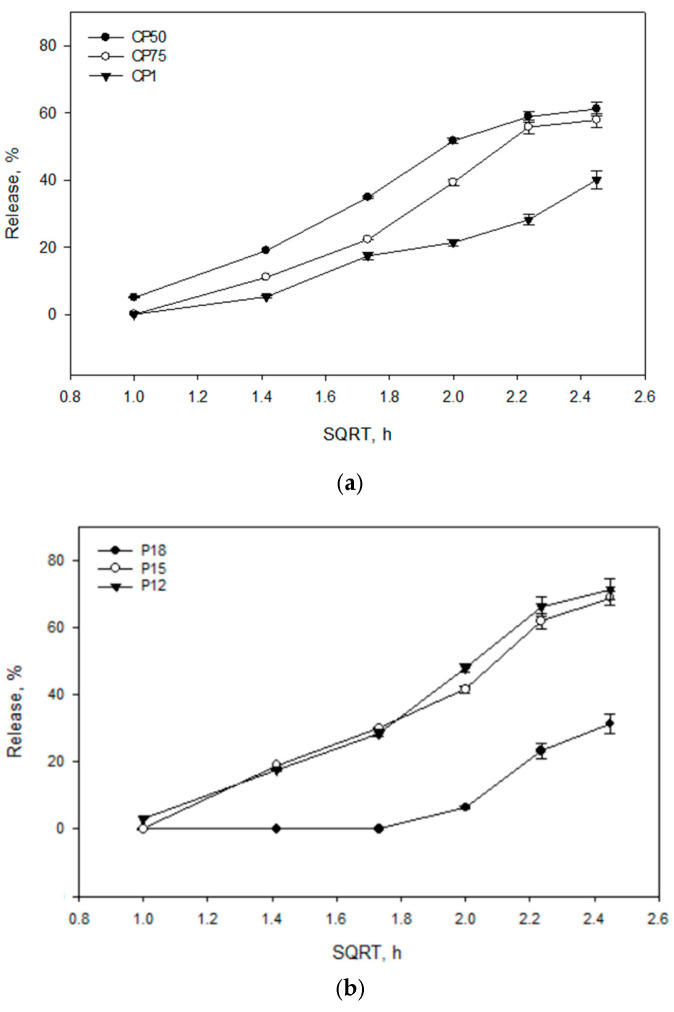

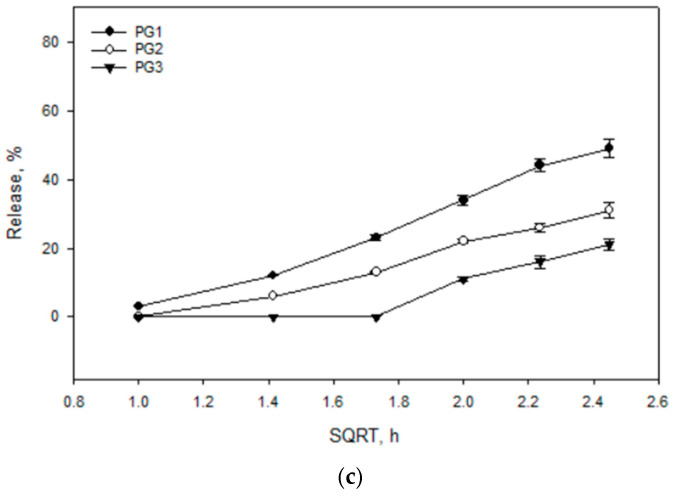
Release kinetics of caffeic acid from gel formulations based on the square root of time (SQRT), following the Higuchi equation. The formulations include (**a**) Carbomer 980-based gels (CP50, CP75, CP1), (**b**) P407-based gels (P12, P15, P18), and (**c**) mixtures of these polymers (PG1, PG2, PG3), (n = 3, mean ± SD).

The results of the study showed that the release rate and efficiency of caffeic acid from the experimental formulations are significantly dependent on the composition of the gelling agents. The carbomer series formulations (CP50, CP75, CP1) (Figure 5a) exhibited a consistent release rate, with caffeic acid release increasing over time. The formulations CP50 and CP75 released the highest amounts of caffeic acid, while CP1 released a statistically significantly lower amount compared to CP50 and CP75 (*p* < 0.05). The P12 formulation (Figure 5b) released the highest amount of caffeic acid, 71.5 ± 3.05%. Other formulations from the P407 series (P15 and P18) achieved 68.90 ± 2.69% and 31.41 ± 2.98% amounts of released caffeic acid, respectively. A statistically significantly slower release of caffeic acid was observed from the P18 formulation containing 18% P407 (*p* < 0.05). Biphasic gels exhibited a slow and consistent release rate. Biphasic formulations released significantly lower amounts of caffeic acid compared to monophase formulations with the same polymer compositions. The PG1 formulation released a statistically significantly lower amount of caffeic acid compared to CP50 and P12 (*p* < 0.05) (Figure 5c). The PG3 formulation released a statistically significantly lower amount of caffeic acid compared to CP1 and P18 (*p* < 0.05). The release results followed the decreasing order: P12; P15; CP50; CP75; PG1; CP1; PG2; P18; and PG3.

**Figure 6:**
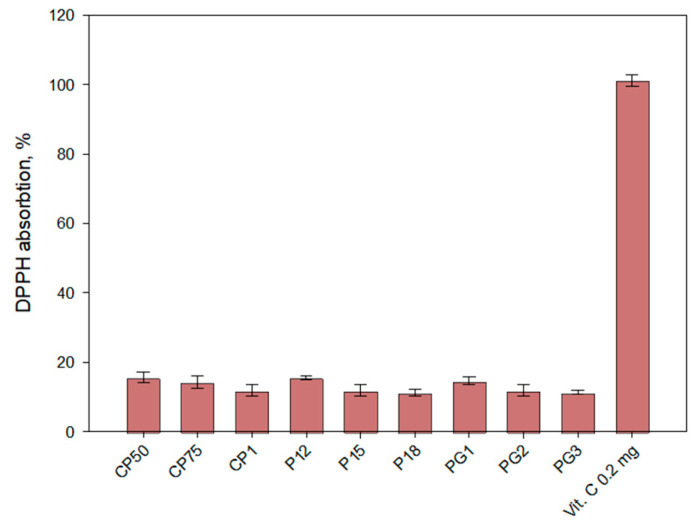
Antioxidant activity of experimental gels in vitro release fractions (acceptor medium) after 6 h by the DPPH method (*n* = 3, mean ± SD).

The antioxidant activity in the gel medium was determined after 6 h of release testing using the DPPH method (Figure 6). The medium exhibited low antioxidant activity, with values ranging from 15.66 to 11.33. The antioxidant activity results were dependent on the viscosity of the gels and their release capacity. The results of antioxidant activity were directly dependent on the amount of caffeic acid released from the formulations.

### 2.4. Experimental Formulations Influence on HaCaT Cell Viability

**Figure 7:**
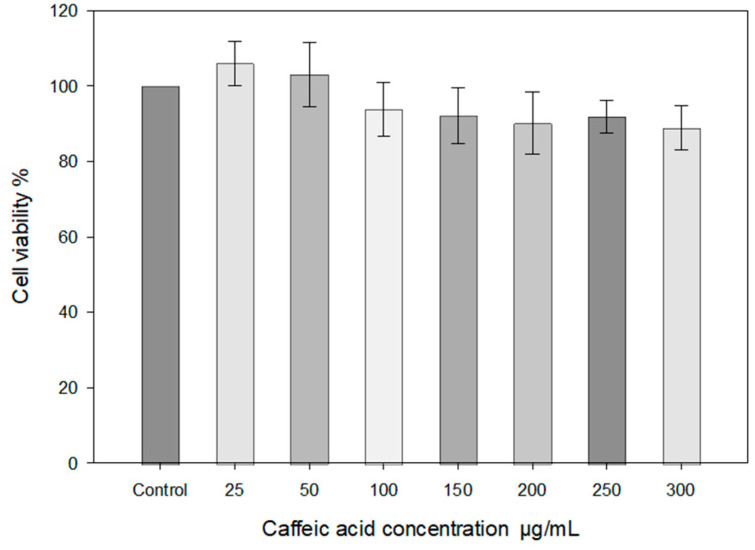
HaCaT cell viability STE test using different concentrations of caffeic acid (medium 30% ethanol). HaCaT cells were treated with different concentrations of caffeic acid (25–300 μg/mL) for 30 min (*n* = 3, mean ± SD).

The caffeic acid solutions (25–300 μg/mL) did not affect the viability of the HaCat cell line, as assessed by cell viability after 30 min (Figure 7). 

**Figure 8:**
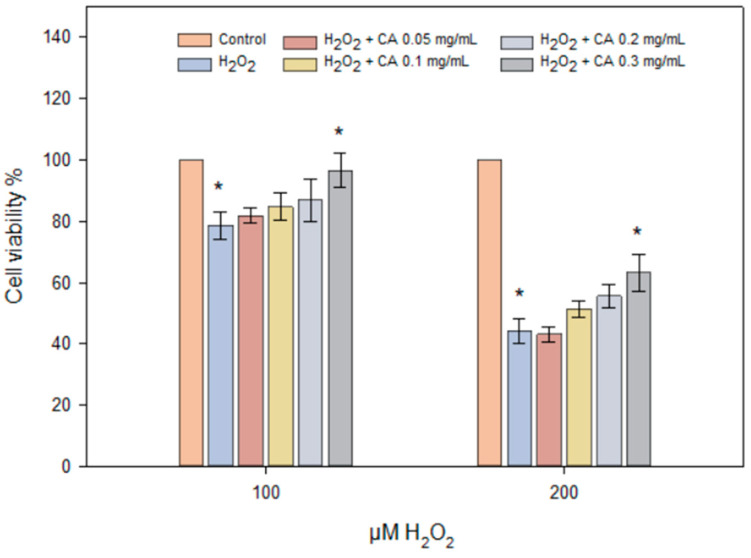
HaCaT cell viability using different concentrations of H_2_O_2_ (100–200 μM) and caffeic acid (0.05–0.3 mg/mL). HaCaT cells were treated with different concentrations of H_2_O_2_ and caffeic acid for 24 h (*n* = 3, mean ± SD). The asterisks (*) indicate statistically significant differences (*p* < 0.05) between the H₂O₂ treatment group and the groups treated with caffeic acid.

In the graph, the viability of HaCaT cells exposed to H₂O₂ (100 μM and 200 μM) along with varying doses of caffeic acid (0.05, 0.1, 0.2, 0.3 mg/mL) is presented (Figure 8). The results indicate that increasing the concentration of H₂O₂ leads to a decrease in cell viability, particularly at the 200 μM concentration. Caffeic acid at 0.3 mg/mL demonstrated a positive effect on cell viability compared to cells affected solely by H₂O₂. The results of the study (Figure 7) showed that higher concentrations of caffeic acid (0.2–0.3 mg/mL) protect cells from damage caused by different concentrations of H₂O₂ and increase cell viability.

### 2.5. Antimicrobial Activity of Experimental Gels

In vitro studies were conducted to determine the antimicrobial activity of caffeic acid using the agar diffusion method. The results are presented in Table 2.

The study aimed to evaluate the antibacterial and antifungal activity of the modeled formulations against *Staphylococcus aureus*, *Escherichia coli*, and *Candida albicans*. All tested formulations demonstrated antibacterial activity against *S. aureus*. The largest inhibition zones were observed for formulations CP50 (18.67 ± 0.58 mm), P12 (18.66 ± 0.58 mm), and P15 (18.17 ± 0.29 mm). These results suggest that these formulations may be effective against Gram-positive microorganisms, such as *S. aureus*. The results showed that the formulations were less effective against *E. coli*, with most inhibition zones being smaller than 5 mm. The antifungal activity tests showed that some formulations exhibited antimicrobial effects against *C. albicans*. The P12 formulation exhibited the largest inhibition zone (11.66 ± 1.15 mm), while formulations CP05 and P15 also showed significant activity with inhibition zones of 10.83 ± 0.76 mm and 10.33 ± 0.58 mm, respectively. All formulations exhibited statistically significantly smaller inhibition zones compared to the chlorhexidine control solution (*p* < 0.05). A direct correlation was observed between the formulations’ ability to release caffeic acid and their antimicrobial activity.

## 3. Discussion

Caffeic acid was selected for this study due to its biological properties, which are suitable for addressing skin aging problems. Scientific studies have demonstrated that the total antioxidant levels in the skin are lower in individuals suffering from atopic dermatitis, psoriasis, and acne compared to those with normal skin [3,33,34]. Treatment with antioxidants, such as ascorbic acid, tocopherol, and polyphenols, is an effective approach to enhance resistance to oxidative stress and delay skin aging [35,36]. In our experiment, the modeled gels are presented as a technological alternative for incorporating caffeic acid into a hydrogel matrix, which can offer long-lasting antioxidant, antimicrobial, and moisturizing effects on the stratum corneum. Currently, there is significant interest in cosmetics derived from natural resources, and within this context, polyphenolic compounds are highlighted for their antioxidant, anti-inflammatory, anti-aging, antimicrobial, and UV-protective effects [37,38,39]. Polymeric hydrogels can respond to changes in pH and temperature in the physiological environment, allowing for the development of systems for prolonged drug deliver [40]. In this study, polymeric systems were formed using different amounts of Poloxamer 407 and carbomer. The research was expected to model stable gelled hydrogel systems with caffeic acid (Table 1). The combination of bioadhesive and thermoresponsive polymers enabled the incorporation of caffeic acid into the modeled carrier systems. Poloxamer 407, due to its reverse thermal gelation properties, has garnered significant interest in the design of extended-release systems. Its solubility-enhancing capability is particularly relevant for addressing the solubility challenges of caffeic acid [29]. The synthetic polymer carbomer has recently been widely utilized as a component in drug delivery systems [41]. In this study, it was selected as a bioadhesive polymer to enhance the gel strength of the P407 hydrogel.

The pH of human skin is between 4.5 and 6, so the pH of products intended for skin application should fall within this range [42]. It can be stated that the pH of the tested gels is within the acceptable range for topical formulations. The incorporation of caffeic acid reduced the pH values of the gels, which is consistent with the literature, as phenolic compounds possess acidic properties [43]. According to published literature, hydrophilic gels containing 0.2% caffeic acid and other components also exhibited antioxidant activity [44]. The results of our study confirmed that hydrophilic gels are suitable for incorporating caffeic acid, as the modeled formulations demonstrated antioxidant and antimicrobial effects. According to the literature, the selected polymers possess important physical and chemical properties and are sensitive to environmental influences, which may be desirable for skin applications, as they allow for the formulation of gels with better contact efficacy, adhesion, extended residence time due to higher retention at the application site, and modified drug release [45].

In our experimental study, the influence of gelling agents and their concentrations on the release of caffeic acid was evaluated (Figure 5). The in vitro release test is a particularly important step in the development of drug delivery systems. For a successful study, it is important to select appropriate experimental conditions that simulate most in vivo conditions [46]. The modeled compositions were tested using magnetic stirring and controlled temperature (32 °C), with a dissolution medium consisting of a water–ethanol mixture to create sink conditions. The dissolution medium was chosen considering the limited solubility of caffeic acid. The results of this study allowed for a comparative evaluation of the release profiles of caffeic acid from different binary hydrophilic gel compositions. The in vitro results of the biopharmaceutical gel study showed that as the concentration of gelling agents increased, and consequently the viscosity of the gel, the amount of active substance released decreased. The results of the study confirmed that an increase in the amount of carbomer can create a more structured system, forming more physical barriers to penetration into the matrix, dissolving the active substance, and transporting it [41,47,48]. The barrier effect of Poloxamer 407 gels on drug release may be due to an increase in the number of water channels, micelle numbers, and micelle size within the gel structure. The shorter intermicellar distance results in more cross-linking between adjacent micelles, leading to increased viscosity and a slower drug release rate [49]. Altuntaş E. et al. (2017) found that a higher concentration of Carbopol 934 in gels containing 18% Poloxamer 407 resulted in a lower release of the active substance (mometasone furoate) [50]. Mathematical analysis of the kinetic profile of caffeic acid showed that regression coefficients of the Higuchi model of gels formulations CP50, CP75, CP1, P12, P15, P18, PG1, PG2, and PG3 were 0.9774, 0.9671, 0.9581, 0.9832, 0.9732, 0.7264, 0.9876, 0.9862, and 0.8115, respectively. The gels modeled by Kevin Garala et al. also exhibited lower release of chlorhexidine hydrochloride at higher concentrations of Poloxamer 407 and Carbopol 934 [49]. Therefore, the results of the in vitro release study of caffeic acid from gels are consistent with the scientific literature regarding the release of active substances from gels containing Poloxamer 407 and carbomer. Gels that released the smallest amount of CA exhibited the weakest antioxidant activity. Caffeic acid may have influenced the kinetics of the DPPH reaction, potentially leading to an underestimation of its antioxidant activity (Figure 6). Compared to the positive control, the amount of caffeic acid released from the formulations exhibited statistically significantly lower antioxidant activity. The presence of polymers can increase the solution’s viscosity, which may slow down the diffusion of caffeic acid and its reaction with the DPPH radical. This can result in a slower reaction rate and an inaccurate assessment of antioxidant activity.

Scientists associate the antioxidant activity of CA with the hydroxyl groups in the structures of phenolic acids and low lipophilicity [51,52]. During the process of inflammation, free radicals are produced [53]. During the study, the antioxidant activity of caffeic acid was investigated on the HaCaT cell culture line under the influence of hydrogen peroxide and varying concentrations of caffeic acid (0.05–0.3 mg/mL), corresponding to the released amount of active substance in vitro (Figure 7). The results demonstrated that caffeic acid at 0.3 mg/mL exhibited statistically significant antioxidant activity compared to the control group, where cells were treated solely with hydrogen peroxide (*p* < 0.05) (Figure 8). Jiyoung Jeon et al. found that caffeic acid reduced the activation of mitogen-activated protein kinases and nuclear factor κB signaling pathways induced by UVB exposure [54].

Currently, in skin treatment and care, particular attention is given to long-acting formulations that ensure a sustained antimicrobial effect and contribute to the healing process [55,56]. The antibacterial activity of caffeic acid is associated with the disruption of bacterial cell functions due to increased acidity of the plasma membrane through proton donation and the acidification of intracellular cytosolic pH; a strongly acidic pH can inhibit the enzyme H+-ATPase, which is required for ATP production [57,58]. The results of our antimicrobial activity study using the agar diffusion method showed that the selected concentrations of caffeic acid solutions inhibited the growth of the tested microorganisms. The results of the antimicrobial study correlated with the ability of the modeled formulations to release caffeic acid.

Ji-Hoon Kim et al. found that caffeic acid and chitosan conjugates are good candidates for developing a therapeutic agent for acne treatment due to their observed antimicrobial effects against *P. acnes*, *S. epidermidis*, *S. aureus*, and *P. aeruginosa* [59]. In this experimental study, it was found that caffeic acid exhibited the strongest effect against *S. aureus* bacteria (Table 2). The results of studies by Mafalda Andrade et al. showed that the antimicrobial activity of CA was directly related to its lipophilicity, which influenced bacterial sensitivity, the physical and chemical properties of bacteria, and membrane integrity. *E. coli* was less sensitive to the effects of compounds compared to *S. aureus* [60]. Our study results also demonstrated similar antimicrobial activities, where *E. coli* was less sensitive to the effects of the compounds than *S. aureus* [60]. Study results confirm that caffeic acid is a potential active substance for designing topical formulations aimed at protecting against oxidative stress and microbiological contamination; 50% ethanol as a control had no effect on the test microbes. According to the scientific literature, the most effective antimicrobial concentration of ethanol is usually between 60 and 90% [61]. In this study, a higher ethanol concentration than that present in the formulations was selected as the negative control. The results indicate that the ethanol content in the formulations does not have an additional effect on the antimicrobial activity of caffeic acid. Such a concentration best damages cell membranes and proteins of microorganisms.

This opens up opportunities for future research to better understand the impact of oxidative stress on the skin and to strategize the therapeutic use of antioxidants.

## 4. Conclusions

The study investigated the application of gelled systems using the gelling agents, carbomer and poloxamer. The chosen strategy of using these gelling agents to model stable hydrophilic systems for the incorporation of caffeic acid proved to be both appropriate and promising. It was found that the appearance and pH values of all gels met the required standards. All gel compositions demonstrated sustained release of caffeic acid (CA) over 6 h. Notably, all the tested gel compositions were capable of releasing caffeic acid following a Higuchi kinetic profile. The correct selection of gelling agents and the amount of caffeic acid allowed the modeling of hydrophilic gels with antimicrobial and antioxidant activity. The results of this study highlight the limitations and opportunities for developing new and diverse research strategies aimed at exploring the effectiveness of hydrophilic gels as carriers for caffeic acid.

## 5. Materials and Methods

### 5.1. Materials

Caffeic acid (Sigma-Aldrich Steinheim, Germany); Poloxamer 407 or Kolliphor P407 (Sigma-Aldrich, Buchs, Switzerland); Carbomer 980 (Lubrizol, Cleveland, OH, USA); Sodium hydroxide (Erba Lachema, Brno, Czech Republic); Ethanol 96 percent (AB Vilniaus degtinė, Vilnius, Lithuania); Purified water (LSMU Laboratory, Kaunas, Lithuania); DMEM—Dulbecco’s Modified Eagle Medium, FBS, Hanks‘ Balanced Salt solution—HBSS, penicillin–streptomycin solution (Gibco, Paisley, UK); DCFH-DA, MTT (Sigma–Aldrich Chemie GmBh, Steinheim, Germany). Scales Scaltec SBC 31 (Scaltec Instruments GmbH, Goettingen, Germany); Magnetic stirrer with heating surface IKA C-MAG HS7 (IKA-Werke GmbH&Co. KG, Staufen, Germany); Viscometer (FUNGILAB, Barcelona, Spain); pH meter Knick pH-meter 766 Calimatic with electrode SE 104 N (Knick Elektronische Meβgerate GmbH&Co, Berlin, Germany); Spectrophotometer with diode array detector Agilent 8453 UV-Vis (Agilent Technologies, Inc., Santa Clara, CA, USA).

### 5.2. Preparation of Gel Bases

#### The Components of Formulated Gels Are Listed in Table 1

Carbomer 980 gels production: Gels were formulated using different concentrations (0.5%, 0.75%, 1%) of Carbomer 980. The respective amount of Carbomer 980 was mixed with purified water. The mixtures were kept at room temperature for not less than 24 h. Gels were neutralized using a 10% sodium hydroxide solution until the pH value reached from 4 to 12, and the mixture was stirred until a homogeneous mass was obtained [32,41].

Poloxamer 407 gels production: Gels were formulated containing 12–18% Poloxamer 407. Gels were prepared using a cold method, where P407 and purified water mixture was kept in a refrigerator (at 4 °C) for not less than 24 h until the poloxamer completely dissolved. The clear liquid mixture of Poloxamer 407 and purified water was stirred until the solution changed to a gel.

Hydrogels of Poloxamer 407 and Carbomer 980 production: gels were formulated containing 12–18% Poloxamer 407 and 0.5% Carbomer 980. The Poloxamer 407 and Carbomer 980 water mixture was kept in a refrigerator (at 4 °C) for not less than 48 h until the poloxamer and carbomer dissolved. The mixture was stirred continuously until it was a homogeneous gel and neutralized using a 10% NaOH solution until the pH was 5.5.

Caffeic acid was dissolved in 96% (*v/v*) ethanol. The dissolved caffeic acid was then incorporated into the gel bases, and the mixture was thoroughly stirred until a homogeneous mass.

### 5.3. Evaluation of the Physicochemical Properties of Gel Bases

A rotational viscometer (Fungilab, Barcelona, Spain) was used to measure the viscosity of polymeric systems. The temperature was controlled at 18 to 37 °C, and the viscometer was programmed to run at a speed of 20 rpm. The rheological properties of the formulations were evaluated with a rheometer (Physica MCR, Anton Paar GmbH, Graz, Austria) using a system of parallel steel plates and a standard-size concentric cylinder geometry. Storage and loss modulus were measured. The measurements were carried out at a temperature from 18 °C to 40 °C, at an angular frequency omega of 1 rad/s, amplitude gamma of 0.5%, and temperature change rate of 2 °C per min. The pH values of gels containing caffeic acid were measured using a Knick 766 Calimatic pH meter with an SE 104 N electrode, suitable for measuring the pH of semi-solid preparations. The pH was measured at 23 ± 1 °C (*n* = 3).

### 5.4. Determination of the Solubility of Caffeic Acid

The solubility study of caffeic acid was conducted using various solvents: purified water, 0.1 N hydrochloric acid solution (pH 1.2), phosphate buffer (pH 6.8), and 30% (*v/v*) ethanol solution. An excess amount of caffeic acid powder was added to test tubes containing the solvents, and the tubes were kept at room temperature for 48 h. After incubation, the solutions were filtered through a double layer of gauze, cotton, and a membrane filter (0.45 µm). The concentration of caffeic acid in the filtered solutions was determined using a UV spectrophotometer (Agilent Technologies, Inc., Santa Clara, CA, USA).

### 5.5. In Vitro Release Study of Caffeic Acid from Formulations

The release of caffeic acid from the formulated gels was studied using modified Franz-type diffusion cells separated by a semi-permeable membrane (Curophan (Medicell International Ltd., London, UK). Before the release test, the membranes were soaked in purified water for 12 h. Then, 1 g of the test gel was placed in the donor compartment. The acceptor compartment was filled with 30 mL of an acceptor medium (a mixture of purified water and 96% ethanol (70:30)); the medium was selected to create sink conditions and enhance the solubility of caffeic acid, which has limited solubility in purely aqueous environments. The temperature of the acceptor medium was maintained at 32 ± 0.5 °C, simulating human skin temperature. The acceptor medium was continuously stirred using a magnetic stirrer with a heating surface (IKA-Werke GmbH&Co. KG, Staufen, Germany). The study was conducted for 6 h. Samples (1 mL) of the acceptor medium were taken at 0.5, 1, 2, 4, and 6 h. A UV-spectrophotometric method was used to determine the amount of caffeic acid. First, a reference solution of caffeic acid was prepared by weighing 2.5 mg of caffeic acid and pouring it into a 25 mL volumetric flask. A mixture of ethanol and purified water (40:60; *v/v*) was added to the flask to achieve a concentration of 100 µg/mL caffeic acid. The surface area of the diffusion membrane separating the donor fluid from the acceptor fluid area was 1.77 cm^2^. The calibration curve was made by preparing solutions of different caffeic acid concentrations, which correspond to 2, 3, 4, 5, 6, 7, and 8 µg/mL caffeic acid concentrations. The acceptor medium was replenished after 4 miming of each sample. The absorbance of each prepared solution was measured with a UV-spectrophotometer at a wavelength of 325 nm [62].

### 5.6. Antimicrobial Activity of Gels with Caffeic Acid Using the Agar Diffusion Method

The antimicrobial activity of caffeic acid gels in vitro was evaluated using the agar diffusion method on Mueller–Hinton agar. Antimicrobial activity was tested against reference strains of *S. aureus* (*ATCC 25923*), *E. coli* (*ATCC 25922*), and *C. albicans* (*ATCC 10231*). The study was conducted according to the standards approved by the Clinical and Laboratory Standards Institute (CLSI) [63]. The microbial inoculum was adjusted approximately 1.5 × 10^8^ CFU/mL before spreading on Mueller–Hinton agar plates. Then, 35 mL of molten Mueller–Hinton agar was poured into Petri dishes. After the medium solidified, bacterial strains were spread on the surface. Wells of 7 mm diameter were made in the agar, and 0.1 mL of the gel samples was added to each well. The plates were incubated for 24 h at 37 °C. The antimicrobial activity of the gel samples with caffeic acid was assessed by measuring the diameter of the inhibition zones (mm) around the wells [64,65]. Chlorhexidine 0.02% solution was used as the positive control; 50% (*v/v*) ethanol and the gel base without caffeic acid (PG0) was used as the negative control to assess whether the antimicrobial effects were solely due to caffeic acid.

### 5.7. Determination of Caffeic Acid Antioxidant Activity Using the DPPH Radical Scavenging Method

For the antioxidant activity assay, a 0.1 mM DPPH solution in 96% ethanol was prepared. For the test, 60 µL of caffeic acid solutions from the release test were mixed with 3 mL of the 0.1 mM DPPH ethanolic solution. The samples were incubated in the dark for 30 min. The absorbance of the test and control samples was measured using an Agilent 8453 UV-Vis spectrophotometer at a wavelength of 518 nm (*n* = 3) [66]; 0.2 mg/mL of ascorbic acid was used as a positive control. The antioxidant activity was expressed as the percentage of inactivated DPPH using the following formula:

$$\mathrm{DPPH},\;\%=\left[\frac{A_{0\;}-{\;A}_{1}}{A_{0}}\right]\times 100\;\%$$

where A_0_ is the absorbance of the control sample, and A_1_ is the absorbance of the test sample [67].

### 5.8. Cell Viability and Measurement of Intracellular ROS

The HaCaT human keratinocyte cell line (CLS Cell Lines Service, 300493) was obtained from Cell Lines Service GmbH (Eppelheim, Germany). Cells were seeded into flasks containing DMEM medium supplemented with 10% fetal bovine serum, 100 U/mL penicillin, and 100 μg/mL streptomycin. The cultures were incubated at 37 °C in an atmosphere of 5% CO_2_ and saturated humidity. The culture was transferred weekly, and the medium was refreshed twice a week.

Twenty-four hours prior to the experiment, cells were seeded into 96-well plates at a density of 30,000 cells per well. Cells were treated with various concentrations of caffeic acid. After 30 min of treatment, cells were washed with PBS, and 180 μL of PBS along with 20 μL of MTT solution was added to each well. Cells were incubated with MTT at 37 °C for 2 h. Following incubation, the dye was removed, and the resulting crystals were dissolved in 100 μL of DMSO per well. Absorbance was measured using a microplate spectrophotometer (Sunrise, Tecan Group Ltd., Männedorf, Switzerland) at 570 nm, with a reference at 620 nm. Results were expressed as a percentage of fluorescence relative to control cells.

ROS levels were assessed using the DCFH-DA dye. Cells were seeded into 96-well plates at a density of 50,000 cells per well. After 24 h, the cells were washed with PBS, and 200 μL of HBSS medium containing 10 μM DCFH-DA was added to each well and incubated at 37 °C for 30 min. Following incubation, the cells were washed with PBS and treated with either caffeic acid and/or H_2_O_2_. Fluorescence was measured using a fluorometer (Thermo Fisher Scientific, Inc., Waltham, MA, USA) with excitation at 488 nm and emission at 525 nm. Data are presented as the mean fluorescence intensity ± SE [68].

### 5.9. Statistical Analysis

Data are presented as mean ± SD of 3 measurements. Statistical analysis was performed using the Kruskal–Wallis test, a nonparametric method, followed by Dunn’s multiple comparison test to evaluate differences between groups. The analysis was conducted using SigmaPlot v. 13.0 (GraphPad Software, Palo Alto, CA, USA) and IBM SPSS Statistics 20 (IBM, Armonk, NY, USA). A value of *p* < 0.05 was considered statistically significant.

## Figures and Tables

**Table 1:** Composition of experimental gels containing caffeic acid, with various concentrations of Carbomer 980 and Poloxamer 407 and their physical appearance.

Formulation Nr.	Caffeic Acid, g	Carbomer 980, g	P407, g	Ethanol (96%), g	Purified Water, g	Appearance
CP50	2	0.5	-	10	ad 100	* 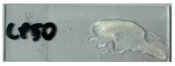 *
CP75	2	0.75	-			* 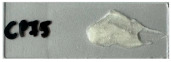 *
CP1	2	1	-			* 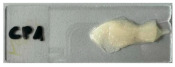 *
P12	2	-	12			* 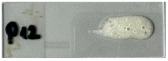 *
P15	2	-	15			* 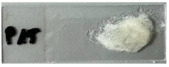 *
P18	2	-	18			* 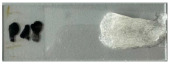 *
PG1	2	0.5	12			* 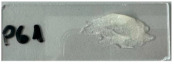 *
PG2	2	0.5	15			* 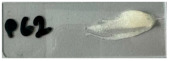 *
PG3	2	0.5	18			* 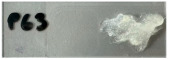 *
PG0	-	0.5	12			

* CP50-1 and PG1-3 gels were neutralized using 10% NaOH solution to the desired pH (range 4–12).

**Table 2:** Antimicrobial activity of gels with caffeic acid using the agar diffusion method (*n* = 3, mean ± SD).

Sample	*S. aureus* Reff.	*E. coli* Reff.	*C. albicans* Reff.
	Zones of inhibition, mm		
CP50	18.17 ± 0.29	<5	10.83 ± 0.76
CP75	17.83 ±1.61	-	8.50 ± 0.50
CP1	11.33 ±1.15	-	8.66 ± 0.58
P12	19.00 ± 0.50	<5	11.66 ± 1.15
P15	18.67 ±0.58	<5	10.33 ± 0.58
P18	9.50 ± 0.50	-	8.33 ± 1.15
PG1	16.33 ± 0.58	<5	7.50 ± 0.50
PG2	10.67 ± 1.26	<5	<5
PG3	6.50 ± 0.50	<5	<5
PG0	-	-	-
50% Ethanol	-	-	-
Chlorhexidine 0.02%	23.67 ± 0.57	20.67 ± 1.53	15.83 ± 0.76

* Activity up to 5 mm is generally marked as <5. PG0 (P407 12%, Carbomer 980 0.5%) gel base without caffeic acid.

## Data Availability

The original contributions presented in this study are included in the article. Further inquiries can be directed to the corresponding author.
